# Supplementary material for: Barriers and Facilitators of Cardiac Rehabilitation in a Middle-Income Country: A Qualitative Study from China
Source: Int J Environ Res Public Health. 2025 Apr 6;22(4):574. doi: 10.3390/ijerph22040574 (PMC12026694; doi:10.3390/ijerph22040574)
Supplement: Supplementary file 1 [file ijerph-22-00574-s001.zip › ijerph-3498636-supplementary.pdf]

S1: Interview Guide

Table S1: Interview Guide for Hospital Directors

Characteristics
- Age
- Gender
- Level of education
- Current occupation

General requirements for rehabilitation
- What type of staff is needed?
- Is there in general sufficient skilled personnel available?

Identifying Barriers and Facilitators
- What barriers and facilitators can you think of for the national implementation of rehabilitation centers?
- What barriers and facilitators are there in the Tianjin/Xi'an area next to the national ones an important factor for the implementation of rehabilitation centers?
- Is rehabilitation affordable for most Urban employees?
  (What can be done to increase the willingness to pay? what can be done to increase the ability to pay?)

Identifying potential strategies
- What is your preferred financial structure of the rehabilitation center?*
- What should be the proper way to include as much as possible patients in the healthcare system?
- What minor reforms could be helpful to organize the rehabilitation for a wider public?
- Given the financial structure of the healthcare system, how could/would the rehab system be structured?
- How could the rehabilitation be reimbursed to maximize the number of attendants?
- Can bonusses in a helpful way be implemented to overcome barriers?
- Which government agencies should be involved to overcome barriers?
- How long can a patient take for a paid sick leave? What determines the duration of sick leave?*

Actual rehabilitation components*
- Do you think it's better for the patients to follow a general program (same to everyone), or a tailored program (disease oriented, health condition or age group oriented)? Please explain why.
- Do you think it's better for the patients to join a group based training or one-on-one training? Please explain why.
- Do you think rehabilitation should focus on physical training (strength, endurance, regaining functioning) or on mental health (coping with the medical problem, peer support, quality of life), or both? Please explain why.

***** *Not reported in this article*

Table S2: Interview Guide for Health Professionals

Characteristics
- Age
- Gender
- Level of education
- Current occupation

Understanding of rehabilitation
- What do you know about rehabilitation?
- Does your patient attend the rehabilitation program? If so, where do they go for rehabilitation? What kind of program was that?
- Are you aware of the difference between western rehabilitation and TCM rehabilitation?*

Attitude towards health outcomes
- What do you think of the statement that "the patient is totally recovered after the operation"? Please explain.
- What do you think of the statement that "the patient has a role in regaining functioning and health situation through training, courses or rehabilitation"? Please explain.
- Do you think rehabilitation will contribute to restore the patients' functioning and health? Why/why not?

Identifying potential strategies
- If you tell your patients that rehabilitation is good for their functioning and health, will they join the rehabilitation program?
- If you tell your colleagues that rehabilitation is good for the patients' functioning and health, will they persuade the patients to join rehabilitation program?
- How do you motivate your colleagues in the advancement of the rehabilitation program?

Actual rehabilitation components*
- Do you think it's better for your patients to follow a general program (same to everyone), or a tailored program (disease oriented, health condition or age group oriented)? Please explain why.
- Do you think it's better for your patients to join a group based training or one-on-one training? Please explain why.
- Do you think rehabilitation should focus on physical training (strength, endurance, regaining functioning) or on mental health (coping with the medical problem, peer support, quality of life), or both? Please explain why.

***** *Not reported in this article*

Table S3: Interview Guide for Rehabilitation Lectures

**Characteristics**<br>- Age<br>- Gender<br>- Level of education<br>- Current occupation
**Knowledge about rehabilitation**<br>- What do you know about rehabilitation?<br>- What kind of rehabilitation program are you teaching?<br>- Are you aware of the difference between western rehabilitation and TCM rehabilitation?*
**Attitude towards health outcomes**<br>- What do you think of the statement that "the patient is totally recovered after the operation"? Please explain.<br>- What do you think of the statement that "the patient has a role in regaining functioning and health situation through training, courses or rehabilitation"? Please explain.<br>- Do you think rehabilitation will contribute to restore the patients' functioning and health? Why/why not?
**Actual rehabilitation components***<br>- Do you think it's better for your patients to follow a general program (same to everyone), or a tailored program (disease oriented, health condition or age group oriented)? Please explain why.<br>- Do you think it's better for your patients to join a group based training or one-on-one training? Please explain why.<br>- Do you think rehabilitation should focus on physical training (strength, endurance, regaining functioning) or on mental health (coping with the medical problem, peer support, quality of life), or both? Please explain why.
**Education in the rehabilitation field**<br>- What type of rehab staffs are trained in your university?<br>- Is there in general sufficient skilled personnel available?<br>- Are there enough qualified teaching staffs in the field of rehab?

***** *Not reported in this article*

Table S4: Interview Guide for Company Employers

**Characteristics**<br>- Age<br>- Gender<br>- Level of education<br>- Current occupation<br>- Which form of your company is? (State-owned, private, foreign invested enterprise, or foreign joint venture)*<br>- How many employees are there in your company?*
**Understanding of rehabilitation**<br>- What do you know about rehabilitation?<br>- Does your employee attend the rehabilitation program? If so, where do they go for rehabilitation? What kind of program was that?<br>- Are you aware of the difference between western rehabilitation and TCM rehabilitation?*
**Attitude towards health outcomes**<br>- What do you think of the statement that "my employee is totally recovered after the operation"? Please explain.<br>- What do you think of the statement that "my employee has a role in regaining functioning and health situation through training, courses or rehabilitation"? Please explain.<br>- Do you think rehabilitation will contribute to restore the patients' functioning and health? Why/why not?
**Identifying potential strategies**<br>- How long can your employee take for a paid sick leave? What determines the duration of sick leave?*<br>- Does the government provide sick leave allowance?*<br>- What can you do to stimulate rehabilitation (so that your employee can resume work more quickly with better function, and less likely of sick leave in the future)?

***** *Not reported in this article*

Table S5: Interview Guide for Patients

**Characteristics**<br>- Age<br>- Gender<br>- Level of education<br>- Current occupation<br>- Primary problem/diagnosis<br>- Will you return to work after discharge?<br>- What is your monthly income?<br>- Who is your informal caregiver?
**Understanding of rehabilitation**<br>- What do you know about rehabilitation?<br>- Do you know someone who attended the rehabilitation program? If so, where do they go for rehabilitation? What kind of program was that?<br>- Are you aware of the difference between western rehabilitation and TCM rehabilitation?*

Attitude towards health outcomes

- What do you think of the statement that “the patient is totally recovered after the operation”? Please explain.
- What do you think of the statement that “the patient has a role in regaining functioning and health situation through training, courses or rehabilitation”? Please explain.
- Do you think rehabilitation will contribute to restore your own functioning and health? Why/why not?

Accessibility to healthcare

- Can you afford the cost of rehabilitation?
- Where do you live?
- How do you get to the rehabilitation center? (Hint: by car, bus, subway, taxi...)
- Do you think the transportation costs a lot of money? How much does it cost for one visit?
- Do you need someone to accompany you to get to the rehabilitation center?
- Do you need an accommodation of short stay in the neighborhood of rehabilitation center, in order to attend the rehabilitation program? (Hint: rent a house/flat, or book a hotel)*

Identifying potential strategies

- If you were told that rehabilitation is good for your functioning and health, are you willing to join the rehabilitation program?
- Who has the strongest power to persuade you? (Hint: government, doctors, families, friends, advertisement, live example of success rehabilitation...)
- Would you be more likely to join rehabilitation program if the costs are covered by insurance? If not, what is the reason?

Actual rehabilitation components*

- Do you want to follow a general rehabilitation program (same to everyone), or do you want to train on your own personal goals? Please explain why.
- Do you want a group based training or one-on-one training? Please explain why.
- Do you think rehabilitation should focus on physical training (strength, endurance, regaining functioning) or on mental health (coping with the medical problem, peer support, quality of life), or both? Please explain why.

*** *Not reported in this article*

Table S6: Interview Guide for Family Members

Characteristics

- Age
- Gender
- Level of education
- Current occupation
- Primary problem/diagnosis of the patient
- Will you return to work after the patient discharge?
- What is your monthly income?
- What is the relationship between you and the patient?

Understanding of rehabilitation

- What do you know about rehabilitation?
- Do you know someone who attended the rehabilitation program? If so, where do they go for rehabilitation? What kind of program was that?
- Are you aware of the difference between western rehabilitation and TCM rehabilitation?*

Attitude towards health outcomes

- What do you think of the statement that “the patient is totally recovered after the operation”? Please explain.
- What do you think of the statement that “the patient has a role in regaining functioning and health situation through training, courses or rehabilitation”? Please explain.
- Do you think rehabilitation will contribute to restore the patients’ functioning and health? Why/why not?

Accessibility to healthcare

- Is the cost of rehabilitation affordable?
- Where do you live?
- How does the patient get to the rehabilitation center? (Hint: by car, bus, subway, taxi...)
- Do you think the transportation costs a lot of money? How much does it cost for one visit?
- Does the patient need your help to get to the rehabilitation center?
- Do you need an accommodation of short stay in the neighborhood of rehabilitation center, in order to support the patient to attend the rehabilitation program? (Hint: rent a house/flat, or book a hotel) *

Identifying potential strategies

- If you were told that rehabilitation is good for the patient’s functioning and health, are you willing to persuade the patient to join the rehabilitation program?
- Who has the strongest power to persuade the patient and you? (Hint: government, doctors, families, friends, advertisement, live example of success rehabilitation...)
- Will the patient be more likely to join rehabilitation program if the costs are covered by insurance? If not, what is the reason?

Actual rehabilitation components*

- Do you think it’s better for the patient to follow a general program (same to everyone), or a tailored program (disease oriented, health condition or age group oriented)? Please explain why.
- Do you think it’s better for the patient to join a group based training or one-on-one training? Please explain why.
- Do you think rehabilitation should focus on physical training (strength, endurance, regaining functioning) or on mental health (coping with the medical problem, peer support, quality of life), or both? Please explain why.

*** *Not reported in this article*

S2: Codebooks

Table S7: Codebook – Barriers

Subthemes and Codes	Example Quotes
Lack of resources	
Lack of resources	Hospital director 4: "The hospitals are not equipped with enough facilities."
Cost burden	Hospital director 1: "I think the cost is a major barrier."
Cost not covered by health insurance	Hospital director 4: "Rehabilitation is not covered by insurance."
Out-of-pocket expense	Health professional 4: "Rehabilitation costs are all at the patients' own expense."
Surgery already cost a lot of money and CR requests additional cost	Family member 2: "For many families, it is already difficult for them to pay for the treatment of the disease. When you tell them to join CR, they are willing to participate, but they are facing financial constraints."
Transportation cost	Family member 1: "It (taxi cost) is expensive."
Lack of trained CR professionals	
Lack of staffs	Hospital director 1: "In this context, the so-called rehabilitation professionals are definitely not enough."
Reason: the rehabilitation discipline is young in China	Hospital director 1: "The rehabilitation discipline is even younger. Therefore, in such a young discipline, the staffs are definitely not enough."
Lack of awareness and acceptance	
Lack of awareness	Health professional 3: "I think the main barrier is the lack of public awareness of rehabilitation."
Lack of acceptance	Hospital director 4: "The patients must believe that rehabilitation is beneficial. But currently very few patients accept it."
Lack of access to CR	
Access to CR	Health professional 1: "There are many factors that prevent them from participating in the program […], the distance from the rehabilitation center, transportation to the rehabilitation center, and whether there is someone who can accompany them to the rehabilitation center."
Lack of coordination	
Coordination barriers	Hospital director 3: "There is no integrated system between general hospital and the rehabilitation institutions. Patient just go to rehabilitation center by themselves."
Low motivation of certain patients	
Certain patients are less motivated	Health professional 2: "Patients who are relatively old, […] or patients with relatively low educational levels, or relatively poor socioeconomic conditions, had lower rehabilitation requirements."

Table S8: Codebook - Facilitators

Subthemes and Codes	Example Quotes
Positive attitude of CR stakeholders	
Positive attitude of Health professionals	Health professional 1: "We are concerned about patients […], (as doctors) we will certainly try to persuade patients to participate in the rehabilitation programs."
Positive attitude of patients	Patient 1: "It is benefit for my health condition, and it is good for my recovery."
Positive attitude of family members	Family member 1: "I will tell him that there are benefits of rehabilitation. Training under the guidance of doctors with a plan, will help him with recovery."
High motivation of certain patients	
Certain patients are more willing to attend CR	Health professional 2: "[..] young patients, or patients with relatively good socioeconomic status, or patients with higher expectation for quality of life, will be more willing to participate in the rehabilitation program."
More awareness	
Awareness and acceptance	Hospital director 2: "I think that rehabilitation will attract more and more attention. The government may have seen the benefit as well. […] It is becoming more and more important. […] and a lot of patients need rehabilitation. […] Rehabilitation does have many good effects."
Government and policy support	
National policy and government support rehabilitation	Health commission 1: "We need rehabilitation in general hospitals and in specialized hospitals. The government will support."
Affordability	Hospital director 3: "Even if it's at their own expenses, they can afford it. In fact, the cost of rehabilitation in relatively low in China."
CR services become available	
CR services become available	Hospital director 4: "In the past two years, many rehabilitation centers have been launched across the country. In big cities, there are very mature procedures."

Table S9: Codebook – Suggestions towards implementing CR

Subthemes and Codes	Example Quotes
Increase the awareness and change perspectives	
Through health education and popularize the knowledge of CR	Hospital director 1: "we need to first increase their awareness of rehabilitation through popularization, publicity, education, and providing some social guidance."
Change perspectives	Health professional 1: "Through health education […], let them realize that surgical treatment or interventional treatment […] is just the beginning of the treatment for the disease. Afterwards, […] rehabilitation also plays a very important role in the early stage after the surgery. This requires a change of their view on the prognosis of the disease. For example, in the case of surgery, the patients believe that

	they will recover to normal levels after surgery, this is not true. They must learn to change their view of it."
Make CR services accessible	
Establish CR in secondary and primary health institutions to make CR accessible	Health professional 1: "For example, communities or community health center (we call it community health center in China), or secondary and primary health institutions, should play some roles. In large communities or residential areas, rehabilitation programs can be established, and community health centers can play a very important role. Because in large communities, residents can go to the nearest community health center for rehabilitation. This solves the problem of inconvenience on transportation to a faraway tertiary hospital like us."
Establish cooperation and coordination	
Coordination and cooperation	Hospital director 4: "The coordination between hospital and community health center is complex and requires more effort."
Medial Alliance	Hospital director 4: "I think the Medical Alliance system is helpful. For example, we (as a tertiary hospital) could provide evaluation and make rehabilitation plan for patients, and they (the patients) could do rehabilitation in the community. Patients could visit our hospital regularly and we could adjust the rehabilitation plan. The cooperation between medical institutions, (through) medical treatment alliance, could be a good solution."
Develop reimbursement methods	
National Health Insurance	Health professional 1: "The most important solution is, if rehabilitation could be included in the National Health Insurance, or if the health insurance could cover most of them, it would greatly help to reduce the financial burden on patients."
Commercial insurance	Hospital director 2: "The social perspective is commercial insurance."
Motivate stakeholders	
Motivate patients:<br>Reimburse the cost of CR by insurance	Family member 2: "In my opinion, if the costs can be covered by health insurance, then more people will be willing to participate in this."
Motivate patients:<br>Let patient know the importance and benefit of CR	Interviewer: "What motivated you to join a rehabilitation program?<br>Patient 1: "It is benefit for my health condition, and it is good for my recovery."
Motivate patients:<br>Peer support	Health professional 4: "I think there is some effect of peer support."
Motivate health professionals:<br>Financial incentives	Health professional 1: "With some financial incentives, physicians will be more willing to referral patients to rehabilitation after treatment."
Motivate health professionals:<br>Let them know the importance and benefit of CR	Health professional 2: "I think the first thing to do is to repeatedly promote the concept of rehabilitation, to let them realize the importance of rehabilitation, and that rehabilitation is beneficial to patients."
Motivate health professionals:<br>Allow the specialists to work at different institutions	Hospital director 2: "In terms of personnel, for example, allow the specialists to work at different institutions. For example, specialist from our hospital can come to community to help."

S3: Checklists

Table S10: Consolidated Criteria for Reporting Qualitative Studies (COREQ): 32-item Checklist

No	Item	Guide questions/description	Check
**Domain 1: Research team and reflexivity**			
Personal Characteristics			
1.	Interviewer/facilitator	Which author/s conducted the interview or focus group?	x
2.	Credentials	What were the researcher's credentials? *E.g. PhD, MD*	x
3.	Occupation	What was their occupation at the time of the study?	x
4.	Gender	Was the researcher male or female?	x
5.	Experience and training	What experience or training did the researcher have?	x
Relationship with participants			
6.	Relationship established	Was a relationship established prior to study commencement?	x
7.	Participant knowledge of the interviewer	What did the participants know about the researcher? e*.g., personal goals, reasons for doing the research*	x
8.	Interviewer characteristics	What characteristics were reported about the interviewer/facilitator? e.g., *Bias, assumptions, the research topic*	x
**Domain 2: Study design**			
Theoretical framework			
9.	Methodological orientation and Theory	What methodological orientation was stated to underpin the study? *e.g., grounded theory, discourse analysis, ethnography, phenomenology, content analysis*	x
Participant selection			
10.	Sampling	How were participants selected? *e.g., purposive, convenience, consecutive, snowball*	x
11.	Method of approach	How were participants approached? e*.g., face-to-face, telephone, mail, email*	x
12.	Sample size	How many participants were in the study?	x
13.	Non-participation	How many people refused to participate or dropped out? Reasons?	x
Setting			
14.	Setting of data collection	Where was the data collected? e*.g., home, clinic, workplace*	x
15.	Presence of non-participants	Was anyone else present besides the participants and researchers?	x

16.	Description of sample	What are the important characteristics of the sample? *e.g., demographic data, date*	x
Data collection			
17.	Interview guide	Were questions, prompts, guides provided by the authors? Was it pilot tested?	x
18.	Repeat interviews	Were repeat interviews carried out? If yes, how many?	NA
19.	Audio/visual recording	Did the research use audio or visual recording to collect the data?	x
20.	Field notes	Were field notes made during and/or after the interview or focus group?	x
21.	Duration	What was the duration of the interviews or focus group?	x
22.	Data saturation	Was data saturation discussed?	x
23.	Transcripts returned	Were transcripts returned to participants for comment and/or correction?	x
**Domain 3: Analysis and findings**			
Data analysis			
24.	Number of data coders	How many data coders coded the data?	x
25.	Description of the coding tree	Did authors provide a description of the coding tree?	*
26.	Derivation of themes	Were themes identified in advance or derived from the data?	x
27.	Software	What software, if applicable, was used to manage the data?	x
28.	Participant checking	Did participants provide feedback on the findings?	x
Reporting			
29.	Quotations presented	Were participant quotations presented to illustrate the themes / findings? Was each quotation identified? e*.g., participant number*	x
30.	Data and findings consistent	Was there consistency between the data presented and the findings?	x
31.	Clarity of major themes	Were major themes clearly presented in the findings?	x
32.	Clarity of minor themes	Is there a description of diverse cases or discussion of minor themes?	x

** Available upon request*

Table S11: A 15-Point Checklist of Criteria for Good Thematic Analysis Process

**Process**	**No.**	**Criteria**	**Check**
Transcription	1.	The data have been transcribed to an appropriate level of detail, and the transcripts have been checked against the tapes for 'accuracy'.	x
Coding	2.	Each data item has been given equal attention in the coding process.	x
	3.	Themes have not been generated from a few vivid examples (an anecdotal approach) but, instead, the coding process has been thorough, inclusive and comprehensive.	x
	4.	All relevant extracts for each theme have been collated.	x
	5.	Themes have been checked against each other and back to the original data set.	x
	6.	Themes are internally coherent, consistent, and distinctive.	x
Analysis	7.	Data have been analyzed rather than just paraphrased or described.	x
	8.	Analysis and data match each other – the extracts illustrate the analytic claims.	x
	9.	Analysis tells a convincing and well-organized story about the data and topic.	x
	10.	A good balance between analytic narrative and illustrative extracts is provided.	x
Overall	11.	Enough time has been allocated to complete all phases of the analysis adequately, without rushing a phase or giving it a once-over-lightly.	x
Written report	12.	The assumptions about TA are clearly explicated.	x
	13.	There is a good fit between what you claim you do, and what you show you have done – i.e., described method and reported analysis are consistent.	x
	14.	The language and concepts used in the report are consistent with the epistemological position of the analysis.	x
	15.	The researcher is positioned as active in the research process; themes do not just 'emerge'.	x
